# Delayed diagnosis of advanced stage Leukemia in Two Pediatric Patients with Oral Manifestation

**DOI:** 10.4317/jced.61067

**Published:** 2023-12-01

**Authors:** Anshul Gangwar

**Affiliations:** 1 Department of Pedodontics and Preventive Dentistry, Institute of Dental Sciences, Bareilly, Uttar Pradesh, India

## Abstract

**Background:**

Leukemia is a disease in which hematopoietic stem cell mutation leads to the uncontrolled growth of immature blood cells. These abnormal cells, which displace healthy cells, are the reason for the bone marrow’s failure and demise.

**Objective:**

To report unnoticed cases of delayed Leukemia in two patients.

**Case Report:**

A 9-year-old male and 14-year-old female born to a non-consanguineous marriage presented with oral manifestations after getting dental treatment in their native place. Acute Leukemia was suspected based on their total blood count.

**Conclusions:**

Understanding the unusual symptoms and signs of acute Leukemia aids in early identification and effective patient care.

** Key words:**Hematologic Neoplasms, Classification, Incidence, Diagnosis, Child, Young Adult.

## Introduction

Leukemia is a diverse range of hematological diseases. It is classified as having either acute or chronic clinical behavior and lymphocytic or myelocytic histologic origin (1).

According to Gunz, Velpeau most likely reported the first instance of Leukemia in 1827. His patient had a distended abdomen, severe hepatosplenomegaly, fever, fatigue, and blood-like gruel. The patient passed away soon after hospitalization. Although the symptoms indicated acute Leukemia, a firm diagnosis could not be made (2).

Rudolf Virchow and John Bennett simultaneously presented the first descriptions of Leukemia in 1845. Benett claimed it is a kind of pymia, a suppuration of the blood leukocypenia, while Virchow claimed it is a disease originating in the tissue-producing blood cells. Virchow coined the term “leukemia” in 1847. The actual cause of Leukemia was finally uncovered in approximately 20 years, corroborating Virchow’s idea (2).

Globally, there were 437,033 cases of Leukemia in 2018; by 2030, it was predicted that there will be 23.6 million cases yearly (3). Various factors, including hereditary and environmental factors, cause childhood leukemia (CL). However, despite numerous attempts across various scientific disciplines, the interplay of the disease’s potential risk factors and causes still needs to be better understood (4).

It might be unusual to encounter leukemia patients with oral symptoms during routine dental practice as an early warning sign. Dental experts made the diagnosis in 25% of cases of acute myelogenous Leukemia and 33% of cases of acute myelomonocytic Leukemia (1,5).

The patient’s previous dentists may have missed the patient’s systemic condition. This case series illustrates the importance of taking a thorough medical history, correlating oral symptoms to systemic illnesses, and time referrals to prevent potentially fatal circumstances.

## Case Report

**Figure 1:**
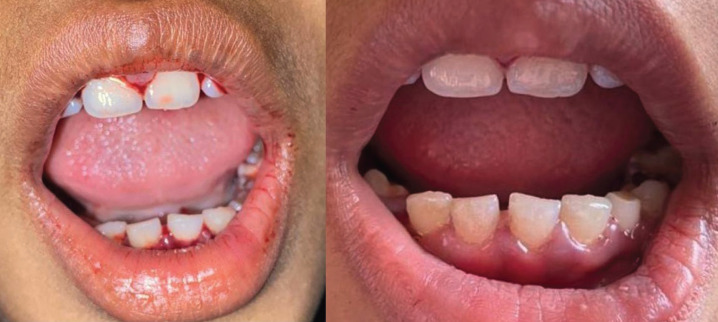
(Patient 1) Intraoral view: a. bleeding from gingiva. b. after administering hemostatic agents- stops bleeding.

Case 1:

A 9-year-old male patient presented to the Department of Paediatric and Preventive Dentistry with a complaint of halitosis for the past two months. The patient was in a mixed dentition period and well-oriented with no history of any bleeding episodes. The palate, tongue, Buccal mucosa, and floor of mouth appeared normal, and no signs of paleness. The Gingiva was haemorrhagic without any indication of hypertrophy (Fig. 1a). Past dental history revealed oral prophylaxis two days back.

Wet gauze was immediately applied as an external pressure pack. Still, it was difficult to control, so the patient was referred to the Pediatrics Department, Rohilkhand Medical College, Bareilly, for systematic evaluations and laboratory blood tests. Tranexamic acid (250mg) was administered intravenously to the patient eight hours X 1day (Fig. 1b). The hematologic profile is presented in Table 1 and consulted with an oncologist.

**Table 1:**
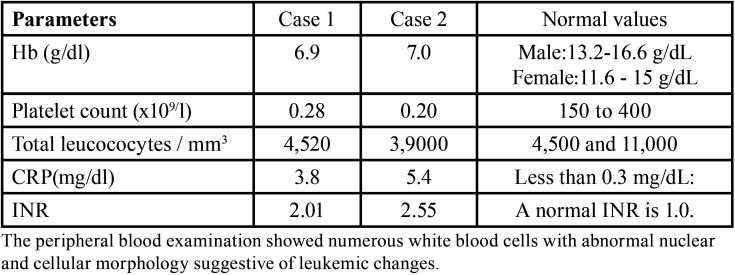
Significant FBC values in case 1 and case 2.

The patient was shifted to the ICU, and a bone marrow biopsy was advised for further confirmatory definitive diagnosis. The patient began experiencing severe headaches, nausea, and leukemic cutis on the third day.

Case 2:

A 14-year-old female patient was brought to the Department with the chief complaint of a bluish-colored lesion in the right lower back tooth region for 5 days.

**Figure 2:**
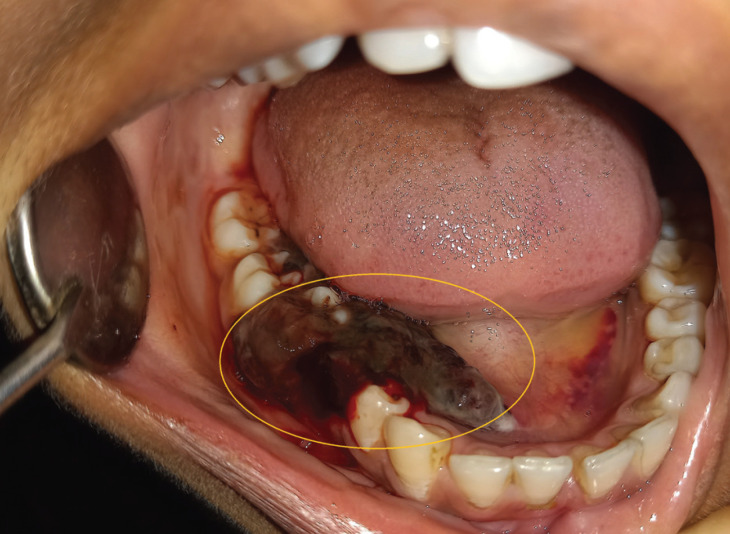
(Patient 2) intraoral lesion wrt right second premolar and molar region (45,46,47).

On intra-oral examination, the hematoma was presented around the right second premolar and molar region (45, 46, and 47), around > 1 cm in diameter both lingually and buccally (Fig. 2). The Gingiva was light pink, and no generalized gingival hypertrophy was seen.

Past dental history- the patient gave a history of extraction of 45 at some private dental clinic.

**Figure 3:**
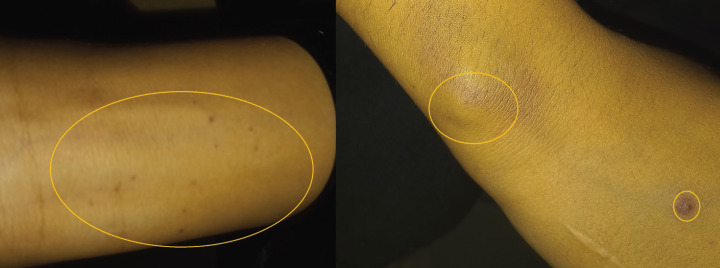
(Patient 2) a. Pin point ecchymosis on the ventral surface of left forearm. b. Bluish nodular lesion on the dorsal surface of right forearm.

Upon general examination, two lesions had been present for five months, with pinpoint ecchymosis on the ventral surface of the left forearm (Fig. 3a) and a bluish nodular lesion on the dorsal side of the right forearm a bluish nodular lesion (Fig. 3b). Additionally, the patient mentioned a bluish spot on her right thigh. The patient wasn’t previously consulted regarding the cutaneous lesions.

The patient was referred to the Hematology Department for a blood investigation (Table 1). The patient was on a soft diet to avoid bleeding aggravation and future treatment for the chief complaint.

## Discussion

The most prevalent malignancy in the world, Leukemia, affected 15.4% of teenagers aged 15 to 19 and 36.1% of children aged 0 to 14 (6).

Around 45,000 children are diagnosed with cancer each year in India, where it is the ninth-place most prevalent killer of children between the ages of 5 and 14 (7). Leukemia’s overall prevalence varied from 26.7 to 52.3% of all CCH. Leukemia prevalence in the CCH population varied from 24.2 to 66.7% for boys to 14.9-50% for girls. The typical annual case count for all forms of Leukemia ranged from 2.7 to 387 (7). The most prevalent type of pediatric malignancy, acute lymphoblastic Leukemia (ALL), accounting for 40-50% of the total burden (8).

Fatigue, anemia, Lymphadenopathy, recurrent infection, bone, stomach, and purpuric pain are some of the systemic clinical symptoms of Leukemia. In 10% of patients, fever is the primary symptom regardless of infection (9).

In suspected cases, a complete blood count is a quick test that can aid in making a precise diagnosis. Bruising, petechiae, and gingival bleeding are a few signs of thrombocytopenia that can occur when the platelet count is below 50,000 cells/mm3 (10,11).

Leukemia cutis, clinically noticeable cutaneous lesions brought on by neoplastic leukocyte infiltration into the epidermis, dermis, or subcutis, is a very uncommon condition that often indicates advanced stage.

The lesions could be mistaken for psoriasis or eczema, among other skin diseases. Herpes simplex, cutaneous mycoses, generalized pruritus, Sweet’s syndrome, pyoderma gangrenosum, exfoliative dermatitis, erythema nodosum, ichthyosis, and vasculitis are among the viruses that can cause these conditions (12).

Depending on the type of Leukemia, its prevalence can range from 3% to 30%, affecting youngsters more frequently. A year following diagnosis, leukemia cutis has a terrible prognosis with an 80% fatality rate (13).

Leukemic cutis was apparent in our second patient at the time of reporting; however, it manifested in our first patient on the third day after admission.

Except for lack of apatite (past 10 days) in patient 1 and fever (sometimes)and petechiae in patient 2 in the current case, other symptoms like fatigue, breathing difficulties, anemia, bone and joint pain, and persistent or recurrent infections were not experienced by either. Both patients/parents forgot to mention their previous dental status. There was no prior history of any medication. Further, the family history has documented no hematologic, genetic, or malignant disorders.

After a brief conversation, both parents were recommended to the higher multispecialty center after being apprised of their patients’ ailments.

Acute Leukemia has an aggressive course if left untreated; mortality usually occurs in under six months (10). With available information from the family sources, both patients were not anymore.

India has made enormous strides in recent decades, but several barriers remain from diagnosis to treatment. A nationally representative study relying on verbal autopsy reports from the Million Death Study estimated childhood cancer mortality at 37/million/year. Only about 4-5% of children received cancer-directed therapy before death, despite 82.5% having visited a hospital before passing. Male deaths outnumbered female deaths by a ratio of 1.6 to 1.14 (14).

## Conclusions

In clinical practice, Leukemia poses a significant obstacle. In some people with acute Leukemia, oral symptoms have been documented as early warning indications. Dentists should carefully screen patients and take appropriate medical histories to reduce the likelihood of medical errors. Early detection and treatment are crucial for the survival of leukemia patients.
